# The social microbiome: gut microbiome diversity and abundance are negatively associated with sociality in a wild mammal

**DOI:** 10.1098/rsos.231305

**Published:** 2023-10-11

**Authors:** Madison Pfau, Sam Degregori, Gina Johnson, Stavi R. Tennenbaum, Paul H. Barber, Conner S. Philson, Daniel T. Blumstein

**Affiliations:** ^1^ Department of Ecology and Evolutionary Biology, University of California, 621 Young Drive South, Los Angeles, CA 90095-1606, USA; ^2^ Rocky Mountain Biological Laboratory, PO Box 519, Crested Butte, CO 81224, USA; ^3^ Department of Ecology and Evolutionary Biology, Princeton University, Princeton, NJ 08544, USA

**Keywords:** social microbiome, microbial diversity, social connectedness, yellow-bellied marmot

## Abstract

The gut microbiome has a well-documented relationship with host fitness. Greater microbial diversity and abundance of specific microbes have been associated with improved fitness outcomes. Intestinal microbes also may be associated with patterns of social behaviour. However, these associations have been largely studied in captive animal models; we know less about microbiome composition as a potential driver of individual social behaviour and position in the wild. We used linear mixed models to quantify the relationship between fecal microbial composition, diversity and social network traits in a wild population of yellow-bellied marmots (*Marmota flaviventer*). We focused our analyses on microbes previously linked to sociability and neurobehavioural alterations in captive rodents, primates and humans. Using 5 years of data, we found microbial diversity (Shannon–Wiener and Faith's phylogenetic diversity) has a modest yet statistically significant negative relationship with the number of social interactions an individual engaged in. We also found a negative relationship between *Streptococcus* spp. relative abundance and two social network measures (clustering coefficient and embeddedness) that quantify an individual's position relative to others in their social group. These findings highlight a potentially consequential relationship between microbial composition and social behaviour in a wild social mammal.

## 1. Introduction

Collective communities of microorganisms in the vertebrate gut, known as the gut microbiome, are pervasive across the animal kingdom [1–3]. Technological advancements in recent years have broadened the potential to study the role microbes play in host physiology and behaviour [5,6]. The symbiotic microorganisms that live in the host's gut, for example, may influence host activity and behaviour, including various aspects of metabolic processes and energy balance [7,8], immune system maturation [9] and nutrient intake [10,11]. Microbial diversity is also associated with Darwinian fitness; improved functional redundancy increases individual disease resilience [12,13]. For example, microbial diversity provides functional redundancy, strengthening an individual's ability to mitigate fitness consequences from the loss or damage to a particular member of the microbial community [14]. These studies demonstrate that a diverse abundance of some microbes in mammalian guts are important to fitness, potentially mediated by neurological and immune function [10,15].

Recent studies identify the brain–gut axis—the communication between the central and enteric nervous systems—as a potential mechanism for the relationship between the gut microbiome, fitness and behaviour [16]. The microbiome has been implicated in stress modulation [9], infant health and metabolism [17], and as a key factor influencing the likelihood of stress-related disorders and diseases [18,19]. In humans, gut microbiome variation can shape personality differences [20], while in non-human animals, studies demonstrate the brain–gut axis's relationship with individual emotions and learning [21] and social behaviour [10,22]. These findings support the concept of the brain gut axis for both social and non-social behaviours.

Social behaviours and group-living have long been associated with fitness through the ability to find mates, acquire food and avoid predators [23–25]. These interactions can influence how populations and territories form and change over time [26]. While studies show that both the microbiome and social behaviour in group-living species individually contribute to fitness, less is known about the gut microbiome–sociality relationship. In a recent study of free-living rhesus macaques (*Macaca mulatta*), a sociality index was related to the abundance of key genera (*Streptococcus* and *Faecalibacterium*; [27]). Studies in rodents have also shown relationships between social connectivity and microbial diversity and abundance [28].

These prior studies have explored the link between the microbiome and sociality in the wild. However, most studies have focused on one causal direction: how social behaviour drives microbiome composition via transmission and acquisition [29–32]. Fewer studies have explored how microbial composition and diversity may influence individual social behaviours [4,33,34]. Given this relationship probably works both ways [35], and given the fitness consequences of sociality and the established role of the brain–gut axis, the microbiome as a driver of sociality may have ecological and evolutionary implications and requires explicit study.

Most studies to date that examined the microbiome as a potential driver of social behaviour have focused on humans or on captive non-human animals (but see [4,33]). In contrast to captive animal systems, wild animals inherit microbial material from a wide range of sources, experience variation in environmental stressors that may be associated with an immune-mediated microbiome response and have higher overall microbial diversity than captive animals [36–39]. These factors can increase microbial diversity and suggest a need for adequate study of the effects of natural drivers of microbial variation and potential associated changes in social behaviour.

We studied associations between microbial diversity and composition with social network attributes in a well-studied population of yellow-bellied marmots (*Marmota flaviventer*) 40]. Marmots are a good natural model to address the sociality–microbiome relationship due to their natural social variability and an available half-decade dataset consisting of individual social observations and microbial samples. Moreover, extensive studies have identified associations between social attributes and marmot fitness (survival: [41–43], alarm call propensity: [44], reproductive success: [45,46], and longevity: [47]), providing a strong foundation for exploring the sociality–microbiome relationship through a structured, exploratory analysis. While the direction of causality is difficult to determine, experimental evidence in laboratory rodent systems shows that changes in gut microbial abundance results in changes in social behaviour (reviewed in [10,16]), bolstering support for studying this direction of this relationship in our wild rodent system.

In marmots, high social connectivity is often associated with reduced fitness: strong affiliative relationships are associated with reduced reproductive success and longevity and a higher likelihood of mortality over the winter hibernation period [43–45,47]. This may be due to yellow-bellied marmots being facultatively social (they may live alone or with others), where most other well-studied social mammals are obligately social, and benefit from group living and increased social connectivity [48].

Gut microbial diversity is associated with gut community stability and increased fitness in other systems [14,49] because it increases resilience against environmental uncertainty [50] and increases immune function [51]. Thus, we initially predicted microbial diversity would be positively associated with sociality in this system because increased heterogeneity and stability may facilitate predictable social behaviours, potentially decreasing social stress [52]. However, given the negative fitness consequences of sociality in our system, an alternative prediction is that gut microbial diversity may relate to decreased social connectivity. Microbial diversity has also been associated with fitness variation in this system. For example, particular microbes such as Firmicutes and lower Bacteroidetes are associated with faster mass gain, an essential fitness correlate of successful hibernation [53]. Given this, we generally predicted that microbes which were associated with increased sociality in other species (table 1) would have a similar relationship in this system. Alternatively, given the fitness costs of increased social connectivity for yellow-bellied marmots (the opposite of most social mammals; [48]), we could generally predict that social-inducing microbes may be negatively associated with sociality in marmots.

**Table 1.** Basic descriptions of the function of each microbe, relationship to social behaviour, and supporting references. Provided to support biological significance of each microbe.

microbe	function	references
**phyla**		
Bacteroidetes	Gram-negative bacteria phylum that is associated with decreased likelihood of neurological disorders and depression	[10,15]
Firmicutes	Gram-positive phylum that is negatively associated with social stress and avoidance, and depressive behaviours	[10,15]
**family**		
Lachnospiraceae	considered a core microbe in adult microbiomes; lack of the family in development is linked to neurodevelopmental disorders	[15,54]
Ruminococcaceae	core microbe: associated with enzyme degradation of plant material; decreased abundance associated with autism in humans	[53,55]
Bifidobacteriaceae	core family, associated with higher dominance behaviours	[56]
Prevotellaceae	significantly enriched in animals exhibiting dominant behaviours	[56]
**genus**		
*Lactobacillus*	modulates inflammatory response; increased *Lactobacillus* reduces stress response and anxiety	[57]
*Clostridium*	social stress correlated with abundance of *Clostridium*; increased abundance also associated with isolation	[10]
*Desulfovibrio*	increased abundance leads to decreased memory, social isolation and impaired learning behaviours	[16]
*Coprococcus*	reduced levels were associated with autism spectrum disorder compared with healthy controls; associated with better life quality	[58]
*Faecalibacterium*	has been associated with upregulation of T cells and anti-inflammatory function	[27,59]
*Sutterella*	more abundant in individuals with higher sociality index, associated with increased direct connectivity in wild social systems	[27]
*Streptococcus*	includes pathogenic strains that can cause disease; associated with less sociality and isolation	[27]
*Prevotella*	more abundant in individuals with higher sociality index; reduced abundance also associated with autism spectrum disorder	[27]
**species**		
*Bacteriodes fragilis*	corrects abnormalities in gut lining and corrects the toxic release of cytokines from other microbes; is associated with decreased instances of autism spectrum disorder in mice	[15]
*Bifiodbacterium longum*	associated with object exploration, memory and decreased anxiety symptoms	[16]
*Streptococcus bovis*	associated with pathology of endocarditis and chronic inflammation; very common pathogenic microbe in animal guts	[60]

Specific *a priori* predictions were developed for microbes in the context of their relationship to sociality from previously identified associations in other species. For example, *Streptococcus* spp. have been negatively associated with a sociability index in wild primates [27] because individuals with pathogenic infections may have less energy to allocate to social interactions. Thus, we also predicted that marmots with higher *Streptococcus* spp*.* abundance will be less social. Further justifications for the development of these *a priori* predictions and their direction can be found in electronic supplementary material, table S1. Given that many microbes, even when examined at the genus and species levels, can have contradictory effects on behaviour, and the facultatively social nature of the yellow-bellied marmot system, these *a priori* predictions are relevant for our system. Other studies should take into account the biology of their system when developing taxon-specific predictions.

## 2. Material and methods

### 2.1. Site specifics and data collection

We studied yellow-bellied marmots from 2015 to 2020 at the Rocky Mountain Biological Laboratory in the Upper East River Valley in Gothic, CO, USA (38°57′ N, 106°59′ W; *ca* 2900 m elevation). Marmots were individually marked and studied at the same colonies annually. Colonies were grouped into two core areas designated as ‘higher elevation' or ‘lower elevation' sites [46]. About 300 m higher, the higher elevation sites experienced harsher weather conditions than the lower elevation areas [61–63].

To uniquely mark individuals and collect fecal microbiome samples, we placed Tomahawk live traps at burrow entrances to capture live marmots. We then transferred trapped individuals to a cloth handling bag and recorded morphological features including body mass, sex, reproductive status and left hindfoot length, before giving each individual a unique metal ear tag (Monel self-piercing fish tags #3, National Band and Tag, Newport, KY) and dorsal fur mark with non-toxic Nyanzol-D dye (Greenville Colorants, Jersey City, NJ) to facilitate identification from afar. To obtain microbiome samples, we collected fecal samples from the traps or during handling which were then placed into a plastic resealable bag and immediately placed on ice before being transferred to a −20°C freezer to await processing [53]. Marmots were in traps for no more than 3 h, meaning fecal samples collected were recent. Fecal matter was cleaned from the traps between use and traps were left in the sun for 6–24 h between use (providing some level of sterilization). If two or more marmots were captured in the same trap at the same time, no fecal samples were collected so as to not misattribute samples to individuals.

We conducted behavioural observations using binoculars and spotting scopes from 20 to 150 m away, distances that limited observer effects on subjects while maximizing the ability to quantify behaviours [64]. We conducted observations during peak marmot activity (7.00–10.00 and 16.00–19.00; [65]), recording and classifying all social interactions as either affiliative (e.g. play, allogrooming) or agonistic (e.g. fighting, chasing). In addition, we recorded the individuals initiating and receiving each interaction as well as the date, time and location of each interaction.

Data were collected under the UCLA Institutional Animal Care and Use protocol (2001-191-01, renewed annually) and with permission from the Colorado Parks and Wildlife (TR917, renewed annually).

### 2.2. Microbiome data processing

Microbiome data collection and analysis followed [53]. Briefly, we isolated bacterial DNA from fecal samples collected from 148 unique individuals using the Qiagen Powersoil Extraction Kit following manufacturer protocols. We generated 16S DNA libraries using the 806R (5'-GGACTACHVHHHTWTCTAAT) and 515F (5'-GTGCCAGCMGCCGCGGTAA) primers targeting the V4 region of the 16S rRNA gene [66]. Target DNA was amplified by PCR using Qiagen Multiplex PCR kits. Following indexing, we sent samples to Laragen (Culver City, CA, USA) for pooling and quantification to create libraries with equimolar sample concentrations. Multiplexed libraries were paired-end sequenced (300 bp per sequence) on an Ilumina Miseq v. 3 at Laragen Sequencing yielded a total of 20 839 221 raw sequencing reads. Overall, sample sequencing depth ranged from 4 reads to 235 203 reads.

We analysed the resulting sequences using QIIME2 (version 2019.9; [67]). First, we imported raw forward and reverse reads and visualized the demultiplexed sequences to determine ideal cut-offs for truncation [67]. Quality control was then completed using the QIIME2 DADA2 denoising tool. All samples were rarefied to a minimum depth of 1000 reads, which yielded a final set of 4 529 579 reads across 286 samples from 148 unique individuals. We summarized the denoised data in a feature table and used this to determine microbial community diversity indices using QIIME2 diversity tools. To examine alpha diversity for downstream analysis in linear mixed models, we calculated Shannon–Wiener diversity and Faith's phylogenetic diversity using a phylogenetic tree generated from the feature table. In order to maintain samples with expected low diversity values, a 1104 sampling depth was implemented in accordance with [53]. We then examined beta diversity at a whole-system level due to its efficiency when analysed comparatively [38,68]. We calculated beta diversity using unweighted UniFrac distances using PERMANOVA with the ‘adonis2' function in the Vegan package (version 2.6-4) for interactions and calculating a PERMANOVA across all samples (999 permutations, performed in QIIME2 v. 2022.2) for main effects and pairwise comparisons [69]. Amplicon sequence variance (ASV) was also used to identify taxonomic assignment at varying levels.

Then, using the *phyloseq* (version 1.38.0) package in R (4.1.3; [70,71]), taxonomic assignments were merged with raw abundance data (the number of reads associated with feature IDs). The data were cleaned to remove phyla of Eukaryota and Cyanobacteria as well the Mitochondria family and Chloroplast order. This was done to ensure that only microbes from marmot hosts were included in the data, not microbes that probably originated from digested plant materials in the fecal samples. Any unassigned phyla were also filtered out of the dataset. For each taxonomic level, the counts across all samples were taken to obtain relative abundance metrics for all runs. Then, centred log ratios (CLR) were performed to remove constraints on the compositional data [72,73]. Any duplicate feature IDs were also removed which yielded a total of 236 observations once paired with the sociality data. We used these values to calculate a weighted average of all microbe abundances in the marmot microbiome. Abundance plots and bacterial association networks with all taxa and their relative abundance were made using the *phyloseq* package in R.

### 2.3. Social network measures

Since marmots share space and burrows with a subset of all individuals at each colony, we defined social groups based on space-use overlap annually (two individuals seen or trapped at the same location and time, or observed using the same burrow, within a one-day interval). Using SOCPROG (version 2.9; [74]), we determined annual simple-ratio pairwise association indices [75] for adults and yearlings. We excluded pups because they emerge halfway through the year and typically interact exclusively with each other and their mother. Association indices were based on the space-use overlap and proportion of time a pair of individuals were seen together each year. We used these indices in the random walk algorithm Map Equation to identify social group membership [76–78]. Map Equation assigns individuals to a single group. However, because social interactions with adult males play a consequential role in marmot social groups by mating with females from multiple matrilines, we added adult males to each group for which they had at least one social interaction with a member of that group [79]. This addition enabled more accurate social network measures to be calculated by maintaining social ties with adult males. Though, for males, network traits were calculated only from their originally assigned group to avoid individual duplicates in the data.

Before constructing networks, we filtered social interactions in two ways. First, we excluded interactions with unknown recipients or initiators to use interactions with only known individuals and direction. While most interactions occurred between identified individuals (81.3% of observed interactions), the initiator and/or recipient could not be identified for some interactions as the interaction was already occurring when the observer arrived or visual obstructions (e.g. marmot orientation to observer, tall vegetation). The exclusion of these undirected interactions between unidentified individuals should not significantly influence social structure [80]. Secondly, we excluded any individuals that were seen or trapped fewer than five times, since these individuals were transients or were dispersing, meaning their interactions were not considered a part of the social group [43–45,47].

With these refined data, we constructed directed and weighted social networks for each year separately based on affiliative interactions using ‘igraph' (version 1.2.11; [71,76]) for each Map Equation defined group in each year. These affiliative networks consisted of 14 093 total social interactions across 66 social groups. We then used social network analysis (SNA) to examine the properties of these social networks. Using SNA, we calculated six social network measures: degree, strength, closeness, eigenvector centrality, clustering coefficient and embeddedness (table 2). The relatively low rate of unknown individuals in our observations, which occurred over the entire active season of these marmots, facilitates the reliability of our social network measures [80,85,86]. We selected each social network measure based on its biological relevance and connection to the individual nature of microbiome data (table 2).

**Table 2.** Descriptions of the six social network measures used to quantify social behaviour and network position. A higher value of each measure corresponds to higher sociality/connectivity.

social measure	description	references
degree	number of social partners an individual has	[64,81]
strength	number of social interactions an individual partakes in, including repeated interactions	[41]
closeness	how centralized an individual is in a network calculated by the reciprocal of the sum of the shortest path lengths between a focal individual and all other individuals in its network	[82,83]
eigenvector centrality	a measure of how connected an individual's social mates are	[41]
clustering coefficient	a measure of an individual's clustering within their network	[84]
embeddedness	quantifies how connected to the group an individual is based on the number of independent links to others in the group	[41,45,64]

### 2.4. Data analysis

We fitted two suites of linear mixed-effects models using *lme4* (version 1.1-30; [87]) to analyse the relationship between social network traits and microbial diversity and abundance. First, we fitted 12 models to quantify the relationship between the six social traits (as the dependent variables) and two microbial diversity measures (Shannon's and Faith's diversity indexes). To control for temporal variance influencing microbial composition, we only included the earliest sample we had for each individual in each year in our analysis. For both Shannon's diversity index and Faith's diversity index, models had 205 observations across 135 unique individuals. Both sets of models consisted of 50 social groups across 5 years (2015–2020). Second, we fitted 102 abundance models for each social trait and each microbe's abundance value. These models had 236 observations consisting of 148 unique individuals in 51 social groups spanning 5 years. The difference in sample size between the diversity and abundance models was due to QIIME2's sampling depth, which reduces sample size for diversity indexes but not for abundance calculations. Clustering coefficient models consisted of 210 observations across 142 unique individuals as clustering coefficient cannot be calculated for certain group sizes (e.g. group of two) or structures (e.g. linear group).

Each social trait was the dependent variable and microbial metrics were included as an independent variable. Additional fixed effects included age class (yearling or adult), valley position (higher or lower elevation), sex and group size. Individual ID and year were fitted as random effects to account for individual and interannual variation. We log_10_ transformed degree, strength, closeness, eigenvector centrality, embeddedness and group size to better meet model assumptions. All continuous variables (the six social traits, group size and all microbial metrics) were standardized (mean-centred and divided by 1 s.d. using the base ‘scale' function in R; [88]). We checked residuals using the *performance* package (version 0.10.2; [89]) to confirm Gaussian assumptions were met. Marginal and conditional *R^2^* values for each model and the semi-partial marginal and conditional *R*^2^ that estimate variance explained by each fixed effect were calculated using the *partR2* package (version 0.9.1; [90,91]). We estimated 95% confidence intervals for our *R*^2^ values using 100 parametric bootstrap iterations. Figures were generated with the R package *ggplot2* (version 3.4.0; [92]) and *sjPlot* (version 2.8.12; [93]).

## 3. Results

### 3.1. Microbiome abundance and distribution

**Figure 1.**
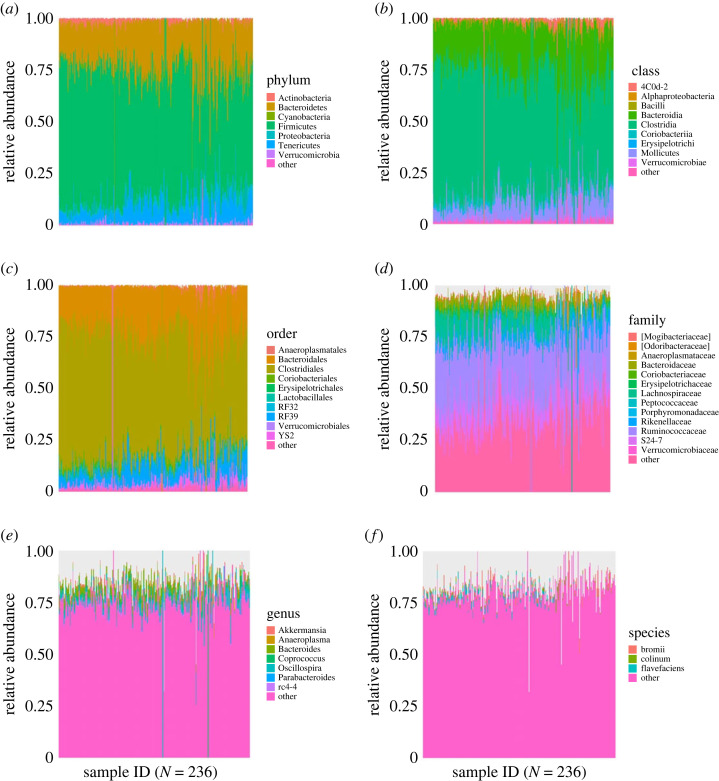
The relative abundance distributions for all taxonomic levels, with sample ID indicating the number of samples and colours corresponding to differing microbial categorizations. The yellow bars accounted for ‘other' categorization. Resolution of microbial taxonomic assignment was reduced at lower taxonomic levels.

At the phyla level, Firmicutes (64.8%) and Bacteroidetes (20.3%) had the highest relative abundance (figure 1*a*) with all other phyla accounting for less than 15%. At the class level, Clostridia and Bacteroidia microbes dominated with 58.3% and 22.5%, respectively (figure 1*c*), and orders within Clostridiales and Bacteroidales accounting for approximately 80.8% of all gut microbes (figure 1*b*). At the family level, Ruminococcaceae had the highest relative abundance (28.8%) followed by Muribaculaceae (14.1%), Lachnospiraceae (6.2%), Bacteroidaceae (3.2%) and Rikenellaceae (2.2%) (figure 1*d*). The remaining were either unassigned family groupings or had significantly lower abundance. Owing to decreasing taxonomic resolution with increasing taxonomic specificity, genus and species-level composition distributions were of low abundance. However, after filtering out unassigned values we found that at the genus level, *Oscillospira* spp*.* accounted for 13.8% of all assigned microbes, *Bacteroides* spp*.* made up 13.5%, *Ruminococcus* spp*.* accounted for 12.2%, and *Coprococcus* spp*. Akkermansia* spp*. Parabacteroides* spp*. Anaeroplasma* spp*.* and *Clostridium* spp. together accounted for the other 23.2% of high abundance microbes assigned (figure 1*e*). At the species level, *Ruminococcus bromii* was the species in highest abundance at 21.1*%, Ruminococcus flavefaciens* and *Clostridium colinum* also had high abundance relative to other assigned microbes, with 19.5% and 11.8%, respectively (figure 1*f*). The skew of microbial distribution towards a select few microbes across different levels and across multiple individuals and social groups suggests low overall diversity of gut microbiota in this wild population of marmots. However, a more even distribution was seen at lower taxonomic levels relative to higher diversity in assigned family, genus and species assignments.

### 3.2. Gut microbe diversity explains variation in some social network measures

**Figure 2.**
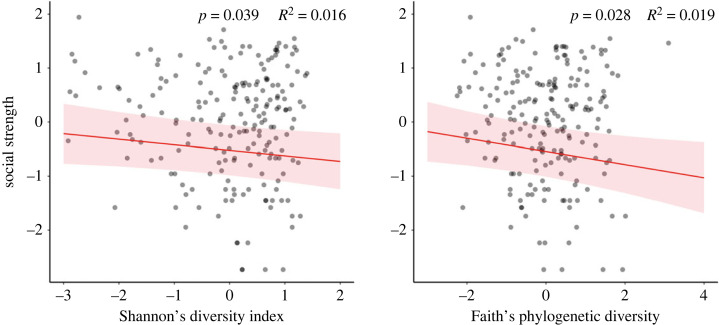
Statistically significant relationships between social strength (number of social interactions) and Shannon's diversity index and Faith's phylogenetic diversity (after controlling for all covariates in the model). Strength was log_10_ transformed and both strength and the two diversity measures were scaled (mean-centred and divided by 1 s.d.; [88]). *p*-value and marginal semi-partial *R*^2^ for each fixed effect are reported.

Alpha microbiome diversity was negatively associated with sociality. Shannon–Weiner diversity was negatively associated with social strength (B = −0.108, *p* = 0.039; figure 2). This model had a marginal *R*^2^ value of 26.0% and a conditional *R*^2^ value of 59.9%. Shannon–Weiner diversity index as a fixed effect alone explained 1.60% of the marginal semi-partial *R^2^* variance, further suggesting that the relationship is relatively modest. Similar to the Shannon–Wiener diversity index, Faith's phylogenetic diversity measure was statistically significant with a weak negative association to strength (*B* = −0.123, *p* = 0.028). This model had a marginal *R*^2^ value of 24.7% and a conditional *R^2^* value of 60.3%. Faith's phylogenetic diversity as a fixed effect explained 1.90% of the marginal semi-partial *R*^2^ variance, again suggesting that diversity, while important, has a modest relationship. Overall, microbial diversity was modestly associated with specific attributes of sociality, namely the number of social interactions an individual participated in (figure 2).

Multivariate analysis of beta diversity via *adonis*2 showed significant differences across feature IDs in individual ID and year, exhibiting variability in diversity among individuals across multiple years. In addition, age class (yearling or adult), sex, valley location (higher elevation or lower elevation), colony area and group size also showed significant differences (table 3). The social network measures embeddedness and eigenvector centrality exhibited a significant difference across feature IDs in beta diversity, suggesting beta diversity associates with variation among individual connectivity and position in their network (table 3).

**Table 3.** Beta diversity results from a PERMANOVA for each measure of sociality, individual attributes (i.e. ID, sex, age class, location) and year. Italic values represent statistical significance (alpha = 0.05).

measure	d.f.	sums of sq.	mean sq.	F-model	*R^2^*	*p*-value
individual ID	158	21.007	0.133	1.089	0.635	*< 0.001*
year	1	0.574	0.574	4.517	0.0174	*< 0.001*
age class	1	0.195	0.195	1.517	0.00594	*0.032*
sex	1	0.488	0.488	3.832	0.0149	*< 0.001*
valley location	1	0.357	0.357	2.790	0.0109	*< 0.001*
group size	14	2.652	0.189	1.511	0.0807	*< 0.001*
colony area	16	2.963	0.185	1.481	0.09	*< 0.001*
degree	23	2.924	0.127	0.986	0.089	0.608
strength	129	16.687	0.129	1.008	0.508	0.392
closeness	74	9.765	0.132	1.035	0.297	0.119
eigenvector centrality	50	8.147	0.163	1.352	0.248	*< 0.001*
clustering coefficient	46	6.089	0.132	1.034	0.185	0.134
embeddedness	8	1.450	0.181	1.425	0.0441	*< 0.001*

### 3.3. Gut microbe abundance explains variation in some social network measures

**Figure 3.**
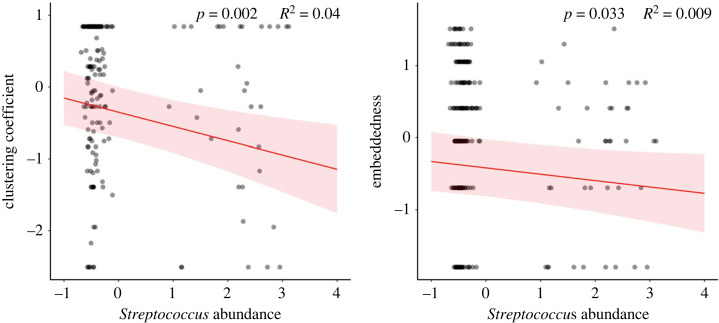
Statistically significant relationships between social position and *Streptococcus* spp. abundance. Clustering coefficient represents if an individual's social partners are also social partners themselves and embeddedness represents how well connected an individual is within the overall structure of the group. Embeddedness was log_10_ transformed and clustering coefficient, embeddedness and *Streptococcus* abundance were scaled (mean-centred and divided by 1 s.d.; [88]). *p*-value and marginal semi-partial *R*^2^ for each fixed effect are reported.

Among our selected microbes (table 1), 16 of the 17 did not show a statistically significant relationship with social behaviour. However, some specific microbial genera had significant relationships to sociality. In particular, *Streptococcus* was negatively associated with two measures of social connectivity, clustering coefficient (*B* = −0.198, *p* = 0.002; figure 3) and embeddedness (*B* = −0.101, *p* = 0.033; figure 3). For the clustering coefficient model, the marginal *R*^2^ value was 16.1% and a conditional *R*^2^ value of 26.6%. *Streptococcus* spp. explained 4.0% of the marginal semi-partial *R*^2^ variance. For the embeddedness and *Streptococcus* abundance model, the marginal *R*^2^ value was 35.6% and a conditional *R*^2^ value of 57.9%. *Streptococcus* spp. as a fixed effect explained 0.9% of the marginal semi-partial *R*^2^ variance, indicating a very modest explanatory value of this microbe to embeddedness.

## 4. Discussion

Although the study of the impacts of sociality on the microbiomes of free-living organisms are in their infancy, recent studies indicate that sociality can shape the diversity and taxonomic composition of gut microbiomes [27,28]. Leveraging a unique long-term dataset on wild marmot colonies, our structured, exploratory analysis showed that approximately 4% of fitted models had statistically significant relationships between features of the intestinal microbiome and an individual's connectivity and position in their social network. These results suggest the gut microbiome has a modest, but in some cases specific and potentially relevant, relationship with an individual's social behaviour in a wild, free-living social mammal population.

The absence of a relationship between social behaviour and the abundance of most microbial taxa may be due to the homogeneity of marmot lifestyles and microbe exposure, particularly within their social groups. Microbe acquisition depends on diet and ingestion of microbe-rich materials [94,95] and marmots experience minimal variation in these key factors [96–98]. Across our study sites, marmots share a similar diet of vegetation and largely remain in the same space, meaning they are not coming across new vegetation often. Within a group, individuals also share burrows and forage in similar locations and thus they may ingest fecal matter. This potential coprophagia may homogenize their gut microbiome, as seen in captive co-housed rodents [99]. This may reduce microbiome variation and limit the ability to detect statistically significant differences in microbial diversity with respect to social behaviour. These results align with previous studies into how captivity affects animal microbiomes, indicating that repeated behaviours and diet homogeneity leads to lower overall microbial variance and diversity, even in wild mammalian systems [100,101]. More mobile species and those with a more diverse diet, like carnivores or omnivores, may have more diverse gut flora [4,33].

Microbial diversity had modest associations with affiliative strength; both Shannon–Weiner's diversity index and Faith's phylogenetic diversity were negatively associated with the social network statistic. As increased microbial diversity is associated with gut stability and increased fitness in other systems [14,49], this result is surprising as increased sociality is typically detrimental to fitness in this system [43,45–47]. As previously stated, this result may be explained by marmots having relatively homogeneous diets and environmental exposure to microbes; as such, increased diversity may be a product of exposure to negative microbes that may reduce energy levels and engagement in sociality. This phenomenon has been observed in other systems where exposure to sickness-inducing microbes and parasites can cause changes in time and energy budgets [102]. Additionally, weighted beta microbial diversity exhibited significant differences in individual ID, year, age class, sex, valley location and group size. Interestingly, individual ID explained the most variation in beta diversity, indicating that each individual marmot varies in microbiome composition. While we observed high microbial homogeneity between individuals overall, these results also indicate that there is observable variance in microbial composition across all individuals, aligning with literature that the microbiome is first and foremost an individualistic metric [94]. Furthermore, group size showed a significant difference across beta diversity, indicating that larger groups differ in microbial diversity to smaller social groups. This may be due to proximity because as group sizes increase, individuals are more likely to engage with similar fecal matter and obtain similar diet materials, indicating they may have more similar and less diverse microbiomes overall than those in smaller groups. For social network statistics, we observed significant differences for eigenvector centrality and embeddedness, with eigenvector centrality being the largest predictor of variance in beta diversity. This shows that social position is associated with variance in beta diversity, suggesting more central individuals, who may use greater space, may have more diverse microbiomes. However, properly evaluating this hypothesis requires additional detailed study.

Notably, these microbial diversity metrics emphasize slightly different aspects of the microbiome. Shannon–Weiner's diversity index calculates diversity based on the number of microbes and their relative abundance. Faith's phylogenetic diversity index considers the number of microbes, but also the number of unique features arising as a function of phylogenetic tree branch length. This difference can yield notable differences, since microbial characterization is not accounted for in estimating diversity using Shannon–Weiner's diversity index [103]. Further, only clustering coefficient and embeddedness were observed to have statistically significant differences in beta diversity, suggesting microbe presence and relative differences may explain some variation in social network position. These results suggest that there is a contributing factor of phylogenetic microbial diversity that may have important consequences for sociality, possibly contributing to fitness in this species [41,42,44–47].

Despite largely no statistically significant relationships between specific microbe's abundance and social network traits, we did observe a relationship with *Streptococcus* spp. abundance. *Streptococcus* spp. has been negatively associated with sociality in wild primates [27]. Here, *Streptococcus* spp. was negatively associated with both clustering coefficient and embeddedness, measures that capture both direct and indirect aspects of social interactions. Interestingly, there are many pathogenic species of *Streptococcus*, and this may suggest health and energy is associated with the ability to engage in social interactions [104]. For example, a marmot that has greater *Streptococcus* spp. abundance may allocate metabolic or cellular resources to immune function, resulting in less available energy resources for social interactions [105,106]. However, in marmots, pathogenic species of this genus (*Streptococcus bovis* and *Streptococcus equi*) were rare. Few individuals had *S. bovis* in their microbiome with individuals within the same years and colony areas exhibiting higher abundance of this species. These results indicate that *S. bovis* may be influencing marmot fitness due to the possible transmission of microbes through closely related individuals. However, further examination of the mode of microbial transmission is necessary to determine the mechanism. Furthermore, because the genus *Streptococcus* contains other opportunistic pathogens that produce inflammation in other species, this may indicate *Streptococcus*'s potential role in modulating physiological stress and potentially further limiting sociality [107,108].

The mechanisms of the brain–gut axis are understudied and their causality is complex. Previous literature has identified some of these difficulties, such as multiple factors influencing microbial content simultaneously [109]. Similarly, identifying the method of how microbial content is shared is difficult in the wild, and especially in burrowing species [28]. For these reasons, we chose to not examine the direction of causality directly, but future work in identifying relationships between social attributes and the microbiome will aid development of formal structural equation models (e.g. [110,111]). Further study is also needed to expand our knowledge of the reverse relationship and casual mechanism: how social behaviour drives microbiome composition in the wild.

Overall, we have shown that microbial variation in a wild populations' gut microbiome may have a modest, but specific and significant, relationship with sociality. Our results suggest gut microbial diversity may modulate sociability through the number of social interactions an individual partakes in, while pathogenic microbes may reduce sociality and influence an individual's position within their social network. This is consistent with previous literature indicating that microbes modulate energy balance and time allocated to engaging in direct social interactions. Further study can follow previous literature by incorporating proximity, kinship and sex in identifying how these microbial relationships across individuals develop and vary [28]. Future studies should also explore more obligately social animals (who largely benefit from increased social interactions, unlike the facultatively social yellow-bellied marmot; [48]), as the role of the microbiome in social behaviour may have a different strength and direction in this system. Symbiotic gut microbes are crucial to the survival of all species and thus understanding the extent and importance of these interactions in the wild is necessary to further understand the causes and consequences of organismal fitness.

## Data Availability

Data and code to replicate these analyses are archived at OSF: http://www.doi.org/10.17605/OSF.IO/F89HR [112]. The data are provided in electronic supplementary material [113].
